# Editorial: Human–robot collaboration in Industry 5.0: a human-centric AI-based approach

**DOI:** 10.3389/frobt.2024.1511126

**Published:** 2024-11-26

**Authors:** Loris Roveda

**Affiliations:** ^1^ Dalle Molle Institute for Artificial Intelligence Research, Lugano, Switzerland; ^2^ University of Applied Sciences and Arts of Southern Switzerland, Manno, Switzerland

**Keywords:** artificial intelligence, human–robot collaboration, human–robot interaction, Industry 5.0, industrial robotics

Industry 5.0 is taking humans at the center of the workspace, avoiding their involvement in non-added value tasks, which can be automatized through the use of robots. In such a way, heavy, onerous, tedious, repetitive operations can be demanded of autonomous systems capable of adapting to the operating conditions, while the operator supervises the process, intervening if necessary. The operators’ expertise is indeed exploited in added-value tasks, enhancing their role.

To effectively implement the Industry 5.0 paradigm, the full potential of human–robot collaboration has to be unleashed. The robot has to efficiently interact with the human, with natural and intuitive communication modalities. It also has to adapt to the requests of the operator, based on the operating environment. To this end, the adoption of AI techniques allows the deployment of intelligent systems capable of perceiving the environment and the humans, embedding reasoning capabilities to make decisions, and learning from the humans and their own experience. These topics are tackled by the papers published in this Research Topic: robots for human assistance in the industrial setting (Dégallier-Rochat et al.), the effects of robotics on humans (Arntz et al.), human–robot collaboration modalities (Mukherjee et al.), and performance evaluation in human–robot collaboration (Remazeilles et al.).

In this Research Topic, the use of AI to enhance human–robot collaboration is indeed investigated. In Dégallier-Rochat et al., the authors discuss how humans can be augmented and not replaced by robotics and AI. The paper focuses on the state-of-the-art human–robot collaboration empowered by AI and its challenges and potential to implement the Industry 5.0 paradigm. Arntz et al. studied the effect on the users of different human–robot interaction modalities. This paper aims to better understand the acceptance and perception operators have of their robotic colleagues. Mukherjee et al. analyzed different human–robot communication modalities, especially concentrating on the naturalness of the interaction. The paper aims to evaluate which interaction approach would be suitable for an effective human–robot interaction in the industry. In Remazeilles et al., the EUROBENCH software framework is introduced to evaluate the performance of bipedal robots, which might be used in the industrial setting to collaborate with humans (i.e., humanoid robots). This paper investigates how to evaluate the performance of such robots in complex scenarios, to better understand how they can assist humans in real workplaces.

In conclusion, this Research Topic provides an overview of current applications of robotics to Industry 5.0, especially making use of artificial intelligence to address the related open issues and challenges. The contributed papers provide interesting approaches to the Research Topic, paving the way to enhanced human–robot collaboration in the industrial setting.

